# Elevated Exhaustion Levels of NK and CD8^+^ T Cells as Indicators for Progression and Prognosis of COVID-19 Disease

**DOI:** 10.3389/fimmu.2020.580237

**Published:** 2020-10-14

**Authors:** Mingyue Li, Weina Guo, Yalan Dong, Xiaobei Wang, Die Dai, Xingxing Liu, Yiquan Wu, Mengmeng Li, Wenjing Zhang, Haifeng Zhou, Zili Zhang, Lan Lin, Zhenyu Kang, Ting Yu, Chunxia Tian, Renjie Qin, Yang Gui, Feng Jiang, Heng Fan, Vigo Heissmeyer, Alexey Sarapultsev, Lin Wang, Shanshan Luo, Desheng Hu

**Affiliations:** ^1^Department of Integrated Traditional Chinese and Western Medicine, Union Hospital, Tongji Medical College, Huazhong University of Science and Technology, Wuhan, China; ^2^Department of Clinical Laboratory, Union Hospital, Tongji Medical College, Huazhong University of Science and Technology, Wuhan, China; ^3^Key Laboratory of Molecular Biophysics of the Ministry of Education, Hubei Key Laboratory of Bioinformatics and Molecular-Imaging, Department of Bioinformatics and Systems Biology, College of Life Science and Technology, Huazhong University of Science and Technology, Wuhan, China; ^4^HIV and AIDS Malignancy Branch, Center for Cancer Research, National Cancer Institute, National Institutes of Health, Bethesda, MD, United States; ^5^Department of Gastroenterology, Henan Provincial People’s Hospital, Zhengzhou, China; ^6^Department of Paediatrics, Tongji Hospital, Tongji Medical College, Huazhong University of Science and Technology, Wuhan, China; ^7^Tianjin University of Traditional Chinese Medicine, Tianjin, China; ^8^Institute for Immunology, Ludwig-Maximilians-Universität München, Munich, Germany; ^9^Institute of Molecular Immunology, Helmholtz Zentrum München, Munich, Germany; ^10^Institute of Immunology and Physiology, Ural Branch of the Russian Academy of Science, Ekaterinburg, Russia; ^11^Institute of Hematology, Union Hospital, Tongji Medical College, Huazhong University of Science and Technology, Wuhan, China

**Keywords:** COVID-19, natural killer (NK) cells, T cells, exhaustion, prognosis

## Abstract

**Background:**

Severe Acute Respiratory Syndrome Coronavirus 2 (SARS-CoV-2) induced Coronavirus Disease 2019 (COVID-19) has posed a global threat to public health. The immune system is crucial in defending and eliminating the virus and infected cells. However, immune dysregulation may result in the rapid progression of COVID-19. Here, we evaluated the subsets, phenotypic and functional characteristics of natural killer (NK) and T cells in patients with COVID-19 and their associations with disease severity.

**Methods:**

Demographic and clinical data of COVID-19 patients enrolled in Wuhan Union Hospital from February 25 to February 27, 2020, were collected and analyzed. The phenotypic and functional characteristics of NK cells and T cells subsets in circulating blood and serum levels of cytokines were analyzed *via* flow cytometry. Then the LASSO logistic regression model was employed to predict risk factors for the severity of COVID-19.

**Results:**

The counts and percentages of NK cells, CD4^+^ T cells, CD8^+^ T cells and NKT cells were significantly reduced in patients with severe symptoms. The cytotoxic CD3^-^CD56^dim^CD16^+^ cell population significantly decreased, while the CD3^-^CD56^dim^CD16^-^ part significantly increased in severe COVID-19 patients. More importantly, elevated expression of regulatory molecules, such as CD244 and programmed death-1 (PD-1), on NK cells and T cells, as well as decreased serum cytotoxic effector molecules including perforin and granzyme A, were detected in patients with COVID-19. The serum IL-6, IL-10, and TNF-α were significantly increased in severe patients. Moreover, the CD3^-^CD56^dim^CD16^-^ cells were screened out as an influential factor in severe cases by LASSO logistic regression.

**Conclusions:**

The functional exhaustion and other subset alteration of NK and T cells may contribute to the progression and improve the prognosis of COVID-19. Surveillance of lymphocyte subsets may in the future enable early screening for signs of critical illness and understanding the pathogenesis of this disease.

## Introduction

Coronavirus disease 2019 (COVID-19), a new type of pneumonia caused by severe acute respiratory syndrome coronavirus 2 (SARS-CoV-2), has broken out in Wuhan, China in December 2019 (1). SARS-CoV-2 is characterized by its strong infectivity and it can be transmitted by asymptomatic patients (2). As of now, COVID-19 has spread to more than 200 countries and regions around the world, more than 1,300,000 people have been confirmed to be infected with SARS-CoV-2 and COVID-19 is associated with nearly 60,000 deaths, and the epidemic could even become more serious in the near future before curative treatments or vaccinations become available.

Little is known about the immunological features in COVID-19 patients. When the viral particles enter the body, the immune system initiates a “battle” against the virus. In the course of this fight it is possible that the immune cells themselves undergo an “education” by the pathogen that includes apoptosis or functional inhibition that will crucially alter disease progression. Thus, it’s critical to identify the immune profiles in COVID-19 patients, which could open up potential intervention strategies and promote further understanding of immunopathology in patients infected with SARS-CoV-2 (3–6). Recent studies showed that the number of T cells and natural killer (NK) cells significantly decreased in severe cases (3–7), and Zheng et al. (3) reported that elevated exhaustion levels and reduced functional diversity of T cells in peripheral blood may predict the disease progression in COVID-19 patients. It is known that NK cells and T cells play a vital role in antiviral immunity. Therefore, there is an urgent need to better characterize and understand the immune status and functionality of these cells in COVID-19 patients.

In this study, we determined differences in the subsets of NK cells and T cells, as well as inflammatory cytokines by flow cytometry in patients with mild and severe illness, as well as healthy volunteers to assess factors associated with the severity of the disease.

## Methods

### Participants

During February 25–February 27, 2020, 32 confirmed COVID-19 patients from Wuhan Union hospital, one of the designated hospitals for the treatment of COVID-19, were included in this study along with 37 age-matched healthy volunteers. Patients’ details, including clinical history, physical examination findings, and routine hematological laboratory results were collected from the medical records to conduct a retrospective analysis. The study protocol was approved by the ethics committee of Tongji Medical College of Huazhong University of Science and Technology. Written informed consent was waived by the Ethics Commission of the designated hospital for the emerging infectious diseases.

Severity of the disease was determined according to the diagnostic and treatment guideline for SARS-CoV-2 issued by Chinese National Health Committee. Briefly, a severe patient was designated when the patient met at least one of the following criteria: 1) Respiratory distress with respiratory frequency of ≥30/min; 2) pulse oximeter oxygen saturation of ≤93% at rest; 3) oxygenation index (artery partial pressure of oxygen/inspired oxygen fraction, PaO2/FiO2) of ≤300 mmHg. According to the criteria above, the enrolled patients were divided into 16 mild and 16 severe cases.

### Respiratory Pathogen Detection

Laboratory validation of SARS-CoV-2 was performed at Wuhan Union Hospital by real-time polymerase-chain-reaction (RT-PCR) as described previously (8). Briefly, throat-swab specimens were obtained from the upper respiratory tract of patients and stored immediately in viral-transport medium. The respiratory sample RNA isolation kit (Zhongzhi, Wuhan, China) was applied to extract the total RNA, which was used to perform the RT-PCR for identifying the virus.

### Blood Collection and Flow Cytometric Analysis

Left-over blood samples for clinical examination from 32 COVID-19 patients and 37 healthy volunteers were collected for analysis. All the samples were processed in the Clinical Lab of Wuhan Union Hospital based on uniform standard procedure. Briefly, peripheral blood mononuclear cells (PBMCs) and plasma were isolated by density gradient centrifugation. Fresh separated PBMCs were stained for flow cytometry, and plasma samples were used for cytokine detection.

Flow cytometry was performed as described previously (9). Briefly, 1 × 10^6^ PBMCs were stained with indicated antibodies in the dark at room temperature for 20 min. After several washes, the cells were analyzed within 1 h. All samples were detected by BD FACS Canto II Flow Cytometry System and analyzed with the BD FACS Diva Software. Antibodies used for flow cytometry included FITC-CD3 (clone: HIT3a), PE-PD-1 (clone: EH12.1), PE/Cy7-CD56 (clone: B159), APC-CD244 (clone: 2-69), APC/Cy7-CD45 (clone: 2D1), BV421-CD16 (clone: 3G8), BV421-CD4 (clone: RPA-T4), PerCP-CD8 (clone: RPA-T8), PE/Cy7-CD27 (clone: M-T271), and all of these were purchased from BD Pharmingen.

### Cytokine Measurement

Plasma cytokines including IL-2, IL-4, IL-10, IL-6, IL-17A, TNF-α, sFas, sFasL, IFN-γ, granzyme A, granzyme B, perforin, and granulysin were measured using Multi-Analyte Flow Assay Kit (740267, Biolegend) by BD FACS Canto II Flow Cytometry System for all patients according to the manufacturer’s instructions.

### Modeling to Predict Risk Factors for Severity of COVID-19

The principal component analysis (PCA) was performed using the ade4 R package. In order to identify immune markers that contribute to the severity of COVID-19, we constructed a classification model using LASSO logistic regression. Firstly, we implemented feature selection using a R package Boruta to find all relevant variables. The selected features were then log_10_-transformed and standardized as z-scores. Data were split into training and test sets for 10 times repeated. Models were validated by 10-fold stratified cross-validation testing and evaluated by calculating the AUROC.

### Statistics Analysis

Normally distributed continuous variables were described as mean and standard deviation (SD), while skewed distribution measures as median and interquartile range (IQR). Correspondingly, student’s t test and Mann–Whitney U test was used to detect the difference. Categorical variables were compared by using Chi-square test or Fisher exact test, which were presented as frequency rates and percentages (%). All statistical analyses were performed using SPSS 13.0 software. *P* value < 0.05 was considered as significant difference.

## Results

### Characterization of Patients With Mild and Severe COVID-19 on Admission

A total of 32 hospitalized patients (including 16 mild cases and 16 severe cases) were enrolled in this study. The detailed characteristics of the patients are shown in **Table 1**. The median age of the patients was 60 years (IQR, 47–68), and 62.5% cases were male. For all the patients, the median time from symptom onset to admission was 15 days (IQR, 7.75–23), and the most common symptoms were fever (68.8%), cough (71.9%), and chest tightness (62.5%), and the rates of comorbidities and oxygen application were 46.9 and 62.5%, respectively. Compared with mild cases, the severe cases showed no significant differences in age, time from symptom onset to admission, fever, and comorbidities but had more male and were more likely to experience cough, chest tightness, and oxygen application. Additionally, consistent with what was reported in other studies (8, 10), severe COVID-19 patients showed significantly higher levels of tissue injury-related enzymes, inflammation related biomarkers, coagulation parameters, and significantly lower levels of nutrition indexes than mild patients (**Table 2**).

**Table 1:** Differences of clinical characteristics between mild and severe COVID-19 patients.

Characteristics	No (%)			
	Total (N = 32)	Mild (N = 16)	Severe (N = 16)	^a^*P*-value
Age, years	60 (47–68)	59 (49–67)	64 (46–78)	0.45
Male	20 (62.5)	7 (43.8)	13 (81.3)	0.03
Hospital admission	15 (7.75–23)	16 (9.5–22.5)	15 (4.25–23)	0.40
Fever	22 (68.8)	10 (62.5)	12 (75)	0.45
Cough	23 (71.9)	8 (50)	15 (93.8)	0.02
Chest tightness	20 (62.5)	6 (37.5)	14 (87.5)	<0.01
Comorbidities	15 (46.9)	6 (37.5)	9 (56.3)	0.29
Oxygen	20 (62.5)	6 (37.5)	14 (87.5)	<0.01

IQR, interquartile range; COVID-19, coronavirus disease 2019. ^a^P values indicate differences between mild and severe patients. P < 0.05 was considered statistically significant.

**Table 2:** Comparison of laboratory parameters between mild and severe COVID-19 patients.

	Median (IQR)			
	Total (N = 32)	Mild (N = 16)	Severe (N = 16)	^a^*P*-value
ALT (U/L)	33 (20–52.5)	24 (19.5-34)	39 (31.8–67.5)	0.04
AST (U/L)	32 (22.5–39)	25 (20.5–33)	38.5 (25–44.3)	0.01
LDH (U/L)	191 (159–215.5)	176 (145–194)	207 (186–230)	0.01
LDL (U/L)	1.4 (1.3–2)	1.3 (1.2–1.4)	1.9 (1.6–2.6)	<0.01
ESR (mm/h)	40.5 (23–70)	23 (20–27.5)	50 (40.5–83.5)	0.01
CRP (mg/L)	3.7 (2.4–19)	2.8 (1.7–8.2)	12.7 (3.7–24)	0.04
FIB (g/L)	4.2 (3.4–5)	3.8 (2.9–4.6)	4.5 (4.2–5.8)	<0.01
FDP(mg/L)	2.8 (1–4.3)	1 (1–3.2)	3.3 (2.2–7.4)	0.03
D-dimer (µg/L)	0.7 (0.4–1.5)	0.4 (0.2–1.3)	1 (0.5–2.4)	0.04
Serum creatinine (µmol/L)	70 (63.4–74)	64 (59–72.8)	73 (68–83)	0.03
Cystatin C (mg/L)	1.2 (1–1.4)	1 (0.8–1.3)	1.3 (1.1–1.5)	0.04
Troponin (µg/L)	1.8 (1.1–2.8)	1.2 (0.9–1.9)	3 (1.6–5.2)	<0.01
Total bile acid (µmol/L)	3.8 (2.8–5.8)	3.3 (1.8–3.7)	5.9 (3.9–11.9)	<0.01
Hemoglobin (g/dL)	121 (112–128)	132 (123–140)	117 (109–125)	0.04
Platelet (10^9^/L)	200 (169–249)	189 (143–200)	233 (171–374)	0.04
IgM (g/L)	56.8 (11.8–85.1)	10.4 (4.9–41.6)	82.8 (56.8–105.6)	0.03

IQR, interquartile range; COVID-19, coronavirus disease 2019; ALT, alanine aminotransferase; AST, aspartate aminotransferase; LDH, lactic dehydrogenase; LDL, low density lipoprotein; ESR, erythrocyte sedimentation rate; CRP, c-reactive protein; FIB, fibrinogen. ^a^P values indicate differences between mild and severe patients. P <0.05 was considered statistically significant.

### Immunologic Features of the Patients With Mild and Severe COVID-19.

The results of blood routine showed that the count of leukocytes in COVID-19 patients significantly increased compared to healthy controls. Regarding the subsets of leukocytes, it is worth to note that the COVID-19 patients showed significantly lower counts of total lymphocyte and basophils, and significantly higher counts of neutrophils, eosinophils, and monocytes upon admission than healthy controls (**Figure S1A**). Moreover, the NK cells and T cells in PBMCs were characterized by flow cytometry. The results showed that percentages and absolute counts of NK cells and T cells in COVID-19 patients were significantly lower than that in healthy controls (**Figure S1B**).

**Figure 1:**
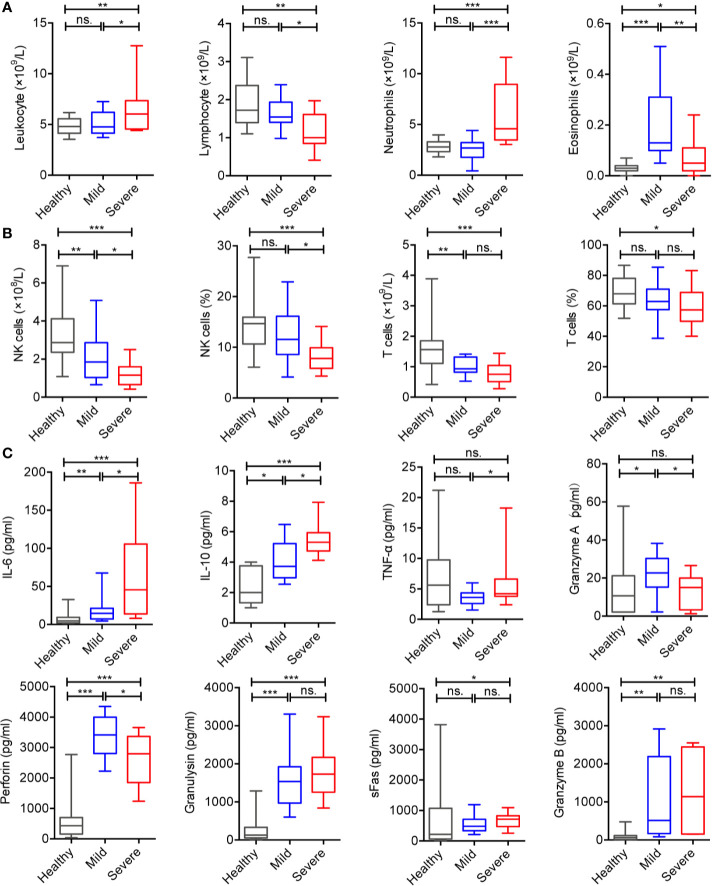
Alteration of immunologic features of the patients with COVID-19. **(A)** Counts of peripheral leukocytes from the healthy controls (n = 37), mild cases (n = 16), and severe cases (n = 16). **(B)** Counts and frequencies of NK cells and T cells from the healthy controls (n = 37), mild cases (n = 16), and severe cases (n = 16). **(C)** Serum cytokines from the healthy controls (n = 37), mild cases (n = 16), and severe cases (n = 16). The level of significance is indicated as follows: ns, not significant; *p < 0.05, **p < 0.01, and ***p < 0.001.

Based on the above changes, we further analyzed these cells in mild and severe cases. We found that, compared with healthy controls or mild patients, the counts of lymphocytes, eosinophils, NK cells, and T cells were significantly lower, while the counts of leukocytes and neutrophils were significantly higher in severe patients (**Figures 1A, B**).

The levels of serum cytokines such as IL-6, IL-10, perforin, granulysin, sFas, and granzyme B in COVID-19 patients were significantly higher than those in healthy controls (**Figure S1C**). In addition, IL-6, IL-10, and TNF-α in severe cases were significantly higher than those in mild cases or healthy controls. Interestingly, the levels of perforin and granzyme A in mild cases were significantly increased compared to severe cases and healthy controls (**Figure 1C**), and no significant differences were observed in IL-2, IL-4, IL-17A, sFasL, IFN-γ, granulysin, sFas, and granzyme B.

### Alteration in the Proportion of NK Cell Subsets and Increased Levels of Regulatory Molecules in Peripheral Blood NK cells

**Figure 2:**
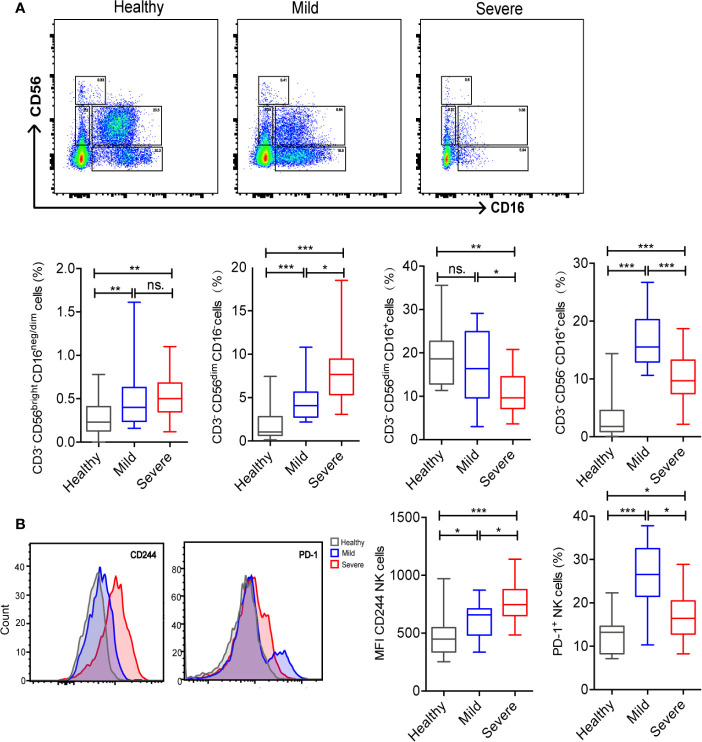
Alteration in the proportion of NK cell subsets and increased levels of regulatory molecules in peripheral blood NK cells from COVID-19 patients. **(A)** Frequency of NK cell subsets out of CD3^-^ cells among groups. **(B)** Comparisons of cell expression modules of regulatory molecules (CD244 and PD-1) in NK cells among groups. The level of significance is indicated as follows: ns, not significant; *p <0.05, **p< 0.01, and ***p<0.001.

As an important part of lymphocytes, NK cells play an indispensable role in innate immunity and can mediate adaptive responses. Our data showed that the count and percentage of NK cells obviously significantly decreased in severe COVID-19 patients. Then, we analyzed the subsets of NK cells. CD16 and CD56 were used to distinguish the subsets of NK cells as described previously (11) (**Figure S2A**). Compared to healthy controls, the COVID-19 patients showed significantly increased percentage of CD3^-^CD56^bright^CD16^neg/dim^ cells, and CD3^-^CD56^dim^CD16^-^ cells (**Figure S2B**). Additionally, the severe patients displayed a significantly lower percentage of CD3^-^CD56^dim^CD16^+^ cells and higher percentage of CD3^-^CD56^dim^CD16^-^ cells than those in mild cases and healthy controls (**Figure 2A**). No difference was observed in the percentage of CD3^-^CD56^bright^CD16^dim/neg^ cells between mild and severe cases (**Figure 2A**).

To further analyze the function of NK cells, we analyzed the immune checkpoints on their surface. Our data indicated that, compared to healthy volunteers, PD-1^+^ NK cells were significantly higher in COVID-19 patients. However, CD244^+^ NK cells showed no significant difference between the two groups (**Figure S2C**). CD244^+^ NK cells were significantly higher in severe patients and PD-1^+^ NK cells were significantly higher in mild cases (**Figure 2B**).

### Imbalanced Proportion of T Cell Subsets and Increased Levels of Regulatory Molecules in Peripheral Blood T Cells

**Figure 3:**
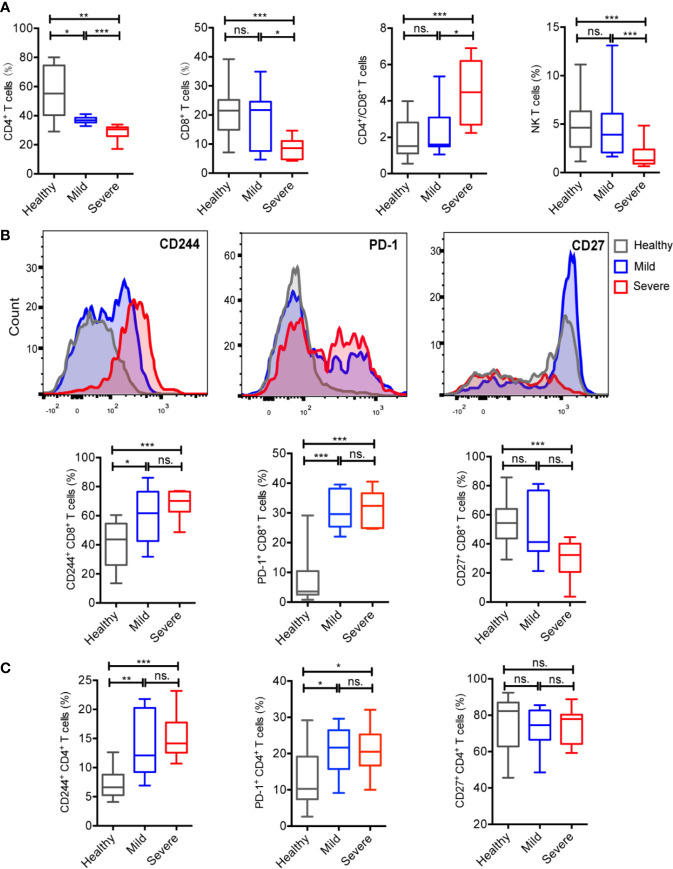
COVID-19 patients showed an imbalanced proportion of T cell subsets and increased levels of regulatory molecules in peripheral blood T cells. **(A)** Percentages of CD4^+^T cells, CD8^+^T cells and NKT cells of total PBMCs and the ratio of CD4^+^/CD8^+^ T cells from the healthy controls (n = 37), mild cases (n = 16), and severe cases (n = 16). **(B)** Comparisons of cell expression modules of regulatory molecules (CD244, PD-1, and CD27) in CD8^+^T cells among groups. **(C)** Comparisons of cell expression modules of regulatory molecules (CD244, PD-1, and CD27) in CD4^+^T cells among groups. The level of significance is indicated as follows: ns, not significant; *p <0.05, **p< 0.01, and ***p<0.001.

T cell subsets, as important members of adaptive immunity, also exhibit certain alterations. In COVID-19 patients, the counts and percentages of CD4^+^ T cells, CD8^+^ T cells and NKT cells were significantly decreased than healthy volunteers (**Figure S3A**). Compared to mild patients, those three T cell subsets also significantly lower in severe patients. Interestingly, the ratio of CD4^+^/CD8^+^ T cells in severe patients was significantly higher than mild patients and healthy controls (**Figure 3A**).

In COVID-19 patients, the CD244 and PD-1 expression levels of CD8^+^ and CD4^+^ T cells were significantly higher and CD27 expression levels of CD8^+^ T cells were significantly lower than healthy controls (**Figure S3B**). Specifically, the CD244 and PD-1 expression levels of CD8+ and CD4+ T cells in both mild and severe patients were significantly higher than healthy controls. In addition, the CD27 expression levels of CD8^+^ T cells in severe patients were significantly lower than healthy controls (**Figures 3B, C**). However, the CD27 expression levels of CD4^+^ T cells showed no significantly differences in among three groups (**Figure 3C**).

### Predictive Effects of Immune Cells and Cytokines on the Severity of Patients

**Figure 4:**
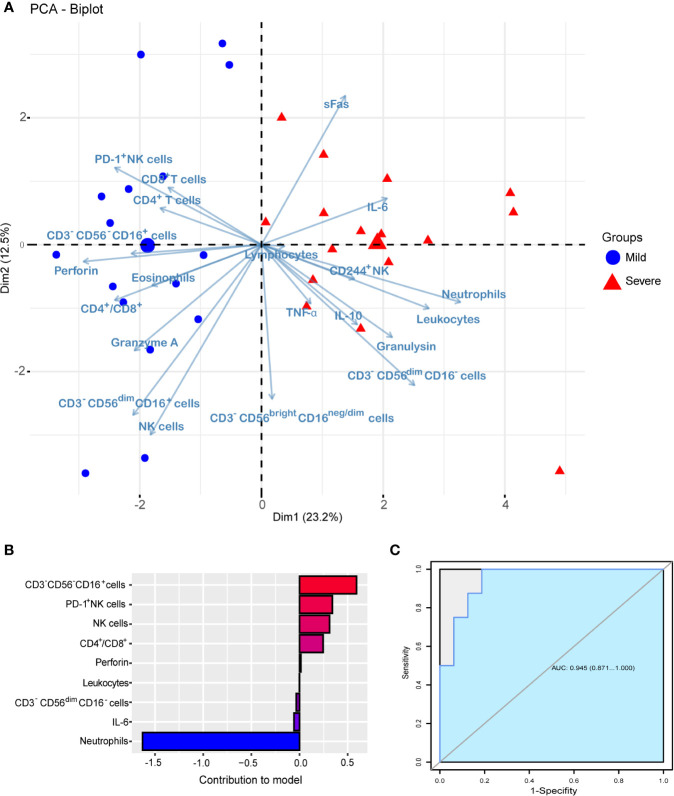
Predictive effect of immune cells and cytokines on the severity of COVID-19 patients. **(A)** PCA analysis of variable distributions for severe (n = 16) and mild (n = 16) patients. **(B)** The top 9 factors show a higher contribution to the severity of COVID-19. **(C)** The AUROC of the LASSO logistic regression model.

In view of the differences in immune cells and cytokines between the mild and severe patients with COVID-19, we further analyzed them to explore the predictive factors for identifying the severity of patients on admission. Firstly, we used the ade4 R package for PCA analysis to identify variable distributions for severe and mild patients (**Figure 4A**). Then, machine learning was performed to screen variables that possess higher contribution to the outcome, and the top nine factors were presented in **Figure 4B**. Neutrophils, IL-6, CD3^-^CD56^dim^CD16^-^ cells and leukocytes were the four mostly influential factors in severe cases, while in mild cases the top five contributing variables were CD3^-^CD56^-^CD16^+^ cells, PD-1^+^ NK cells, NK cells, CD4^+^/CD8^+^, and perforin. However, CD3^-^CD56^-^ CD16^+^ cells could contain rare CD14^+^ CD19^+^ cells, and it would be better to exclude the CD14^+^ CD19^+^ cells in future investigations. Finally, the selected variables were used in LASSO modeling to predict the severity of COVID-19 patients. To evaluate the predictive power of the model built from these indicators, we analyzed the receiver operating characteristic (ROC) curve and calculated the area under ROC curve (AUROC), and the AUROC was 0.945 (**Figure 4C**). Together, these data illustrate the usefulness of measuring the subsets of NK cells at an early stage of hospitalization to determine the development of the disease.

## Discussion

**Figure 5:**
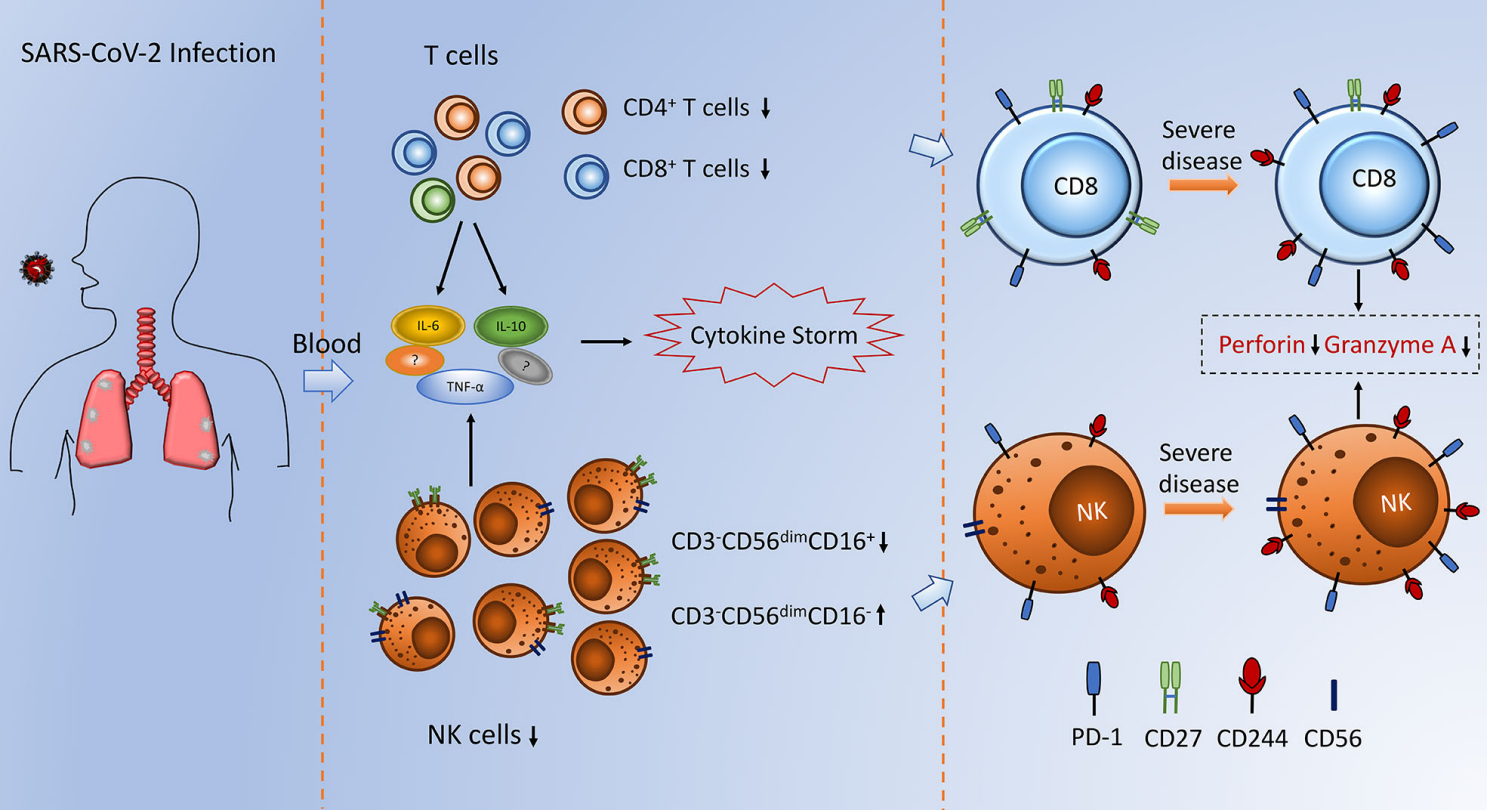
Schematic depiction of alterations in NK cells and T cells observed during SARS-CoV-2 infection.

Viruses can cause a series of immune responses after entering into the human body, and understanding the characteristics of the immune response is of great significance in preventing and treating infectious diseases. However, the profiles of the immune system after SARS-CoV-2 infection are not fully understood currently. This study focused on the alteration of lymphocyte subsets in mild and severe patients with COVID-19, and attempted to explore the relationship between the alterations in the immune system and disease severity (**Figure 5**).

To mount an antiviral response, innate immune cells need to detect the invasion of the virus by various receptors, including those recognizing pathogen-associated molecular patterns (PAMPs). This recognition event leads to the activation of a downstream signaling cascade, i.e. NF-κB and IRF3, accompanied by their nuclear translocation. In the nucleus, these transcription factors induce expression of type I IFN and other pro-inflammatory cytokines and these initial responses comprise the first line defense against viral infection at the entry site (12). Subsequently, the Th1 type immune response plays a dominant role in an adaptive immunity to fight against viral infections. Cytokine microenvironment generated by antigen presenting cells dictates the direction of T cell responses. To be specific, helper CD4^+^T cells orchestrate the overall adaptive response, while cytotoxic CD8^+^T cells are essential in killing of viral infected cells (13).

In the cases of COVID-19, a robust immune response may help the host to clear the virus. However, the excessive activation of immune system is harmful for human body. A “cytokine storm” followed by strong inflammation can initiate widespread tissue damage, such as inflammatory-induced lung injury and other complications including pneumonitis, acute respiratory distress syndrome (ARDS), respiratory failure, shock, organ failure and potentially death (8).

In line with other reports (8, 10, 14, 15), we found the hallmarks of severe cases are neutrophilia, lymphopenia, elevated inflammatory factors, and hypercoagulable states. Specifically, the increase in hyperinflammatory neutrophils yields deteriorating consequences to the infected host that manifests in lung immunopathology, including pneumonitis or ARDS (13). In fact, increased neutrophil cell numbers are consistently observed in the severe or lethal cases of SARS-CoV or MERS-CoV infection (16, 17).

NK cells are innate lymphoid cells, which can directly kill infected cells and contribute to the activation and orientation of adaptive immune response (18). Human NK cells can be subdivided into various subsets based on the relative expression of CD16 and CD56. CD3^-^CD56^bright^CD16^neg/dim^ cells are considered efficient cytokine producers endowed with immunoregulatory properties (19), whereas CD3^-^CD56^dim^CD16^+^ cells are essentially cytotoxic (20). In this study, we extensively characterized the changes in four NK cell subsets and evaluated the phenotypic characteristics of NK cells in COVID-19 patients. In line with another study of COVID-19 (20), our data revealed that the count and frequency of NK cells is significantly lower in severe cases when compared to mild cases and healthy volunteers, which indicates that a decreased NK cell numbers are associated with an increased COVID-19 severity. It’s noteworthy that compared to mild cases and healthy volunteers, the frequency of cytotoxic CD3^-^CD56^dim^CD16^+^ cells decreased significantly in severe cases, which can partially explain why these patients fail to control viral replication.

Programmed death-1 (PD-1) and CD244 are immunoregulatory receptors found on many immune cell types, including NK cells and T cells, and that represent potential therapeutic targets (21, 22). Accumulating research has linked PD-1 and CD244 inhibitory signaling to the maintenance of an exhausted phenotype in NK cells and T cells in chronic infection and cancer (22–26). Our study found that the expression levels of PD-1 and CD244 on NK cells are significantly elevated in patients with COVID-19 compared to healthy volunteers, which signifies an exhausted state of NK cells in patients with COVID-19.

T cells play a vital role in the adaptive immunity against viral infections. As a member of coronavirus, it has been reported that SARS-CoV directly infects macrophages and T cells, which is a key feature in SARS-CoV-mediated pathogenesis (16). However, whether SARS-CoV-2 infects any immune cells is still unknown. In our study, we found that the counts and frequencies of CD4^+^T cells, CD8^+^ T cells, and NKT cells were significantly lower in severe cases than mild cases. More importantly, the significantly elevated expression levels of PD-1 and CD244 on CD8^+^ T cells indicate that SARS-CoV-2 induces T cell exhaustion in COVID-19 patients. Furthermore, our data revealed that the expression of CD27 on CD8^+^ T cells of severe cases is significantly lower than mild cases. As a co-stimulatory immune-checkpoint receptor, CD27 is constitutively expressed on a broad range of T cells, and co-stimulation of CD8^+^ T cells through CD27 promotes immune activation and enhances primary, secondary, memory and recall responses towards viral infections (27). Therefore, the reduction of CD27 expression may impede CD8^+^ T cells activation, which is consistent with the exhaustion of CD8^+^ T cells.

According to a recent report analyzing 138 hospitalized patients with COVID-19 high plasma concentrations of cytokines and chemokines including IL-2, IL-7, IL-10, G-CSF, IP-10, MCP-1, MIP-1A, and TNF-α were observed in the COVID-19 severe cases (15). In addition, another early study has shown that increased amounts of proinflammatory cytokines in the serum (e.g. IL-6 and IL-12) were associated with pulmonary inflammation and extensive lung damage in SARS patients (28). In our study, we also found that, compared to mild cases, the serum levels of IL-10, TNF-α and IL-6 were significantly higher in the severe cases, indicating that severe cases are prone to form an inflammatory storm, leading to rapid deterioration of COVID-19 eventually.

NK cells and cytotoxic T lymphocytes (CTL) play essential roles in protecting the organism against intracellular pathogens, like viruses. As key effector molecules of NK cells and CTLs, perforin, and granzyme are toxins, stored within secretory granules, which exocytose their contents in response to immune synapse formation between these effector cells and virus-infected cells (29). It is worth noting that the serum levels of perforin and Granzyme A were significantly lower in severe cases compared to mild cases, which revealed that the cytotoxic effector cells are dysfunctional in severe patients with COVID-19, and the phenomenon further suggested that the ability of host to control viral replication was decreased.

Since our study revealed the changed immune profile in patients with COVID-19, we wanted to figure out whether changes in the immune system can predict the severity of the disease. Interestingly, we demonstrated the high potency of using immune cells and cytokines as a risk predictor for identifying the severe patients. And among all variables, neutrophils, IL-6, CD3^-^CD56^dim^CD16^-^ cells and leukocytes were the four most influential factors in severe cases, while in mild cases the first five contributing variables were CD3^-^CD56^-^CD16^+^ cells, PD-1^+^ NK cells, NK cells, CD4^+^/CD8^+^ and perforin. Furthermore, neutrophils contributed the most in this model, which is consistent with the stronger inflammation response in the severe “patients” with COVID-19, and it also illustrates the usefulness of measuring the subsets of NK cells at an early stage of hospitalization to determine the development of the disease.

In conclusion, the SARS-CoV-2 infection might affect lymphocytes primarily, especially NK cells and T lymphocytes, resulting in a significant decrease in percentage, changes in subpopulations and elevated expression levels of immune checkpoints, which may be associated with disease severity. Together with clinical characteristics, immunologic indicators including diminished lymphocyte subpopulations and elevated expression level of checkpoints may serve as potential markers for prognosis in COVID-19. Thus, surveillance of clinical characteristics and lymphocyte subsets is helpful for the early screening of critical illness and understanding the pathogenesis of COVID-19.

## Data Availability Statement

The raw data supporting the conclusions of this article will be made available by the authors, without undue reservation.

## Ethics Statement

The studies involving human participants were reviewed and approved by ethics committee of Tongji Medical College of Huazhong University of Science and Technology. The ethics committee waived the requirement of written informed consent for participation. Written informed consent was not obtained from the individual(s) for the publication of any potentially identifiable images or data included in this article.

## Author Contributions

DH, SL, and LW designed the study. MiL, WG, YD, XW, DD, MeL, and WZ researched the data. WG, YD, and MiL contributed to the data analysis. LL, ZK, TY, CT, YG, RQ, and ZZ contributed to the discussion. WG, YD, HZ, MiL, and DH wrote the manuscript. HF, FJ, LW, SL, YW, VH, AS, and DH reviewed/edited the manuscript. All authors contributed to the article and approved the submitted version.

## Funding

This study was funded by the grants from the National Natural Science Foundation of China (nos. 31770983 and 81974249 to DH and no. 81601747 to SL).

## Conflict of Interest

The authors declare that the research was conducted in the absence of any commercial or financial relationships that could be construed as a potential conflict of interest.

## Supplementary Material

Supplementary Figure 1 The comparison of immunologic features between healthy volunteers and patients with COVID-19. **(A)** Counts of peripheral leukocytes from the healthy controls (n = 37) and patients with COVID-19 (n = 32). **(B)** Counts and frequencies of NK cells and T cells from the healthy controls (n = 37) and patients with COVID-19 (n = 32). **(C)** Serum cytokines from the healthy controls (n = 37) and patients with COVID-19 (n = 32). The level of significance is indicated as follows: ns, not significant; *p <0.05, **p< 0.01, and ***p<0.001.

Supplementary Figure 2 COVID-19 patients showed alteration in the proportion of NK cell subsets and increased levels of regulatory molecules in peripheral blood NK cells. **(A)** Definitions of CD3^-^CD56^bright^CD16^neg/dim^ (I), CD3^-^CD56^dim^CD16^-^ (II), CD3^-^CD56^dim^CD16^+^ (III) and CD3^-^CD56^neg^CD16^+^ (IV) cells by flow cytometry from one representative participant. **(B)** Frequency of NK cell subsets out of CD3^-^ cells in healthy controls (n = 37) and patients with COVID-19 (n = 32). **(C)** Comparisons of cell expression modules of regulatory molecules (CD244 and PD-1) of NK cells in healthy controls (n = 37) and patients with COVID-19 (n = 32). The level of significance is indicated as follows: ns, not significant; *p <0.05, **p< 0.01, and ***p<0.001.

Supplementary Figure 3 COVID-19 patients showed an imbalanced proportion of T cell subsets and increased levels of regulatory molecules in peripheral blood T cells. **(A)** Percentages of CD4^+^T cells, CD8^+^T cells and NKT cells of total PBMCs and the ratio of CD4^+^/CD8^+^ T cells from the healthy controls (n = 37) and patients with COVID-19 (n = 32). **(B)** Comparisons of cell expression modules of regulatory molecules (CD244, PD-1 and CD27) in CD8^+^T and CD4^+^T cells in healthy controls (n = 37) and patients with COVID-19 (n = 32). The level of significance is indicated as follows: ns, not significant; *p <0.05, **p< 0.01, and ***p<0.001.
