# Supplementary figures and images for: Elevated Exhaustion Levels of NK and CD8+ T Cells as Indicators for Progression and Prognosis of COVID-19 Disease
Source: Front Immunol. 2020 Oct 14;11:580237. doi: 10.3389/fimmu.2020.580237 (PMC7591707; doi:10.3389/fimmu.2020.580237)

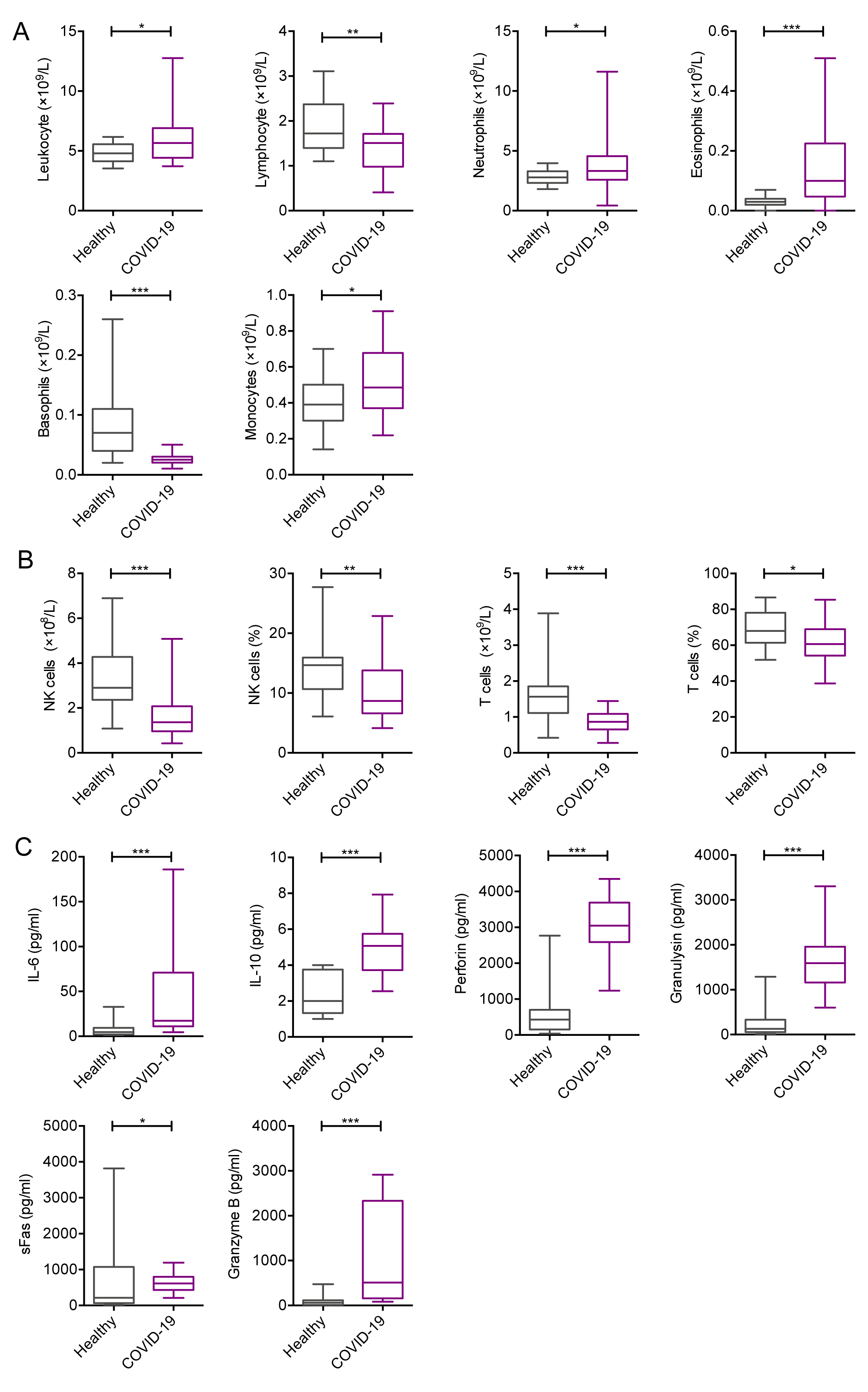

Supplement: Supplementary Figure 1 — The comparison of immunologic features between healthy volunteers and patients with COVID-19. (A) Counts of peripheral leukocytes from the healthy controls (n = 37) and patients with COVID-19 (n = 32). (B) Counts and frequencies of NK cells and T cells from the healthy controls (n = 37) and patients with COVID-19 (n = 32). (C) Serum cytokines from the healthy controls (n = 37) and patients with COVID-19 (n = 32). The level of significance is indicated as follows: ns, not significant; *p <0.05, **p< 0.01, and ***p<0.001. [file Image_1.tif]

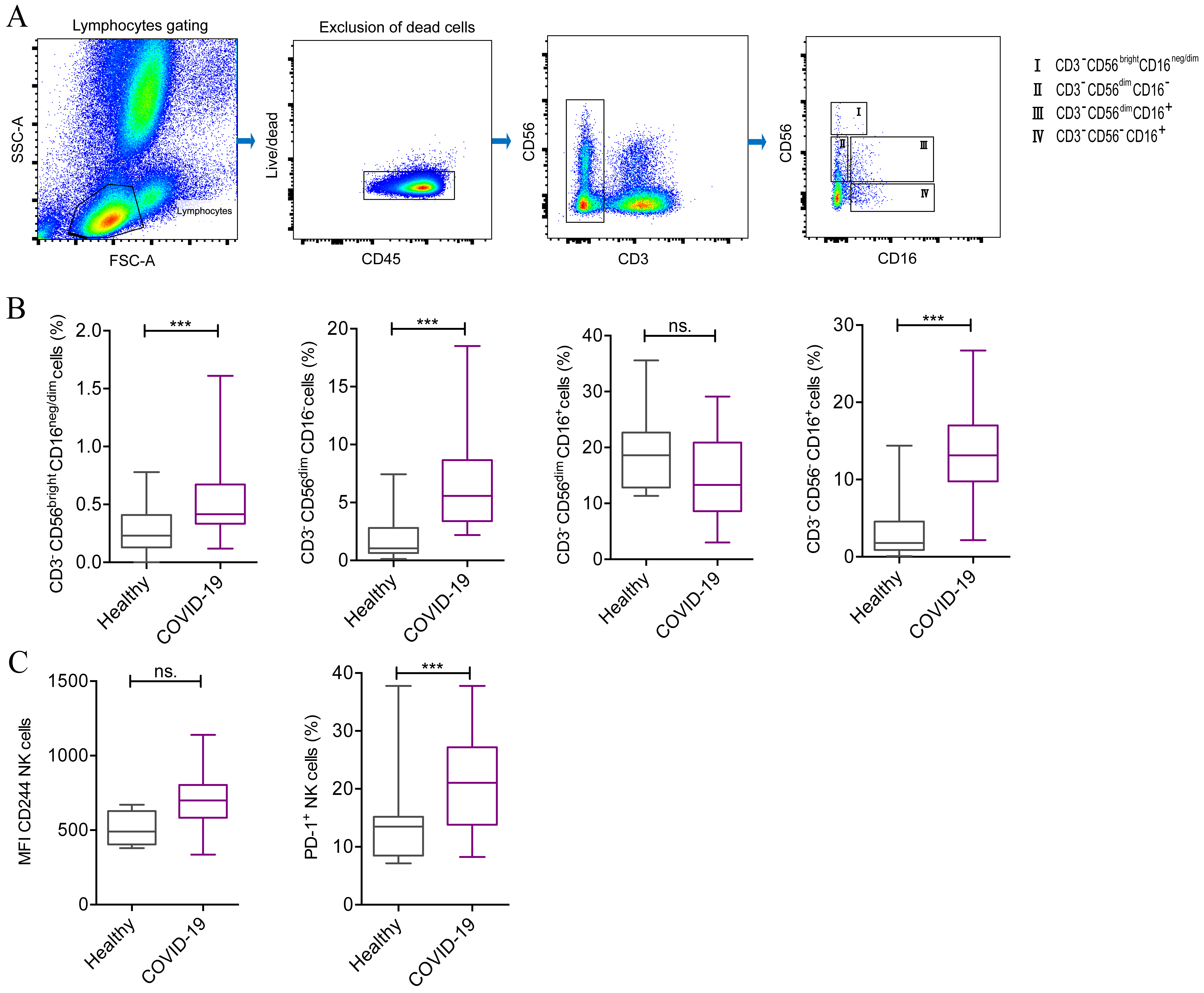

Supplement: Supplementary Figure 2 — COVID-19 patients showed alteration in the proportion of NK cell subsets and increased levels of regulatory molecules in peripheral blood NK cells. (A) Definitions of CD3-CD56brightCD16neg/dim (I), CD3-CD56dimCD16- (II), CD3-CD56dimCD16+ (III) and CD3-CD56negCD16+ (IV) cells by flow cytometry from one representative participant. (B) Frequency of NK cell subsets out of CD3- cells in healthy controls (n = 37) and patients with COVID-19 (n = 32). (C) Comparisons of cell expression modules of regulatory molecules (CD244 and PD-1) of NK cells in healthy controls (n = 37) and patients with COVID-19 (n = 32). The level of significance is indicated as follows: ns, not significant; *p <0.05, **p< 0.01, and ***p<0.001. [file Image_2.tif]

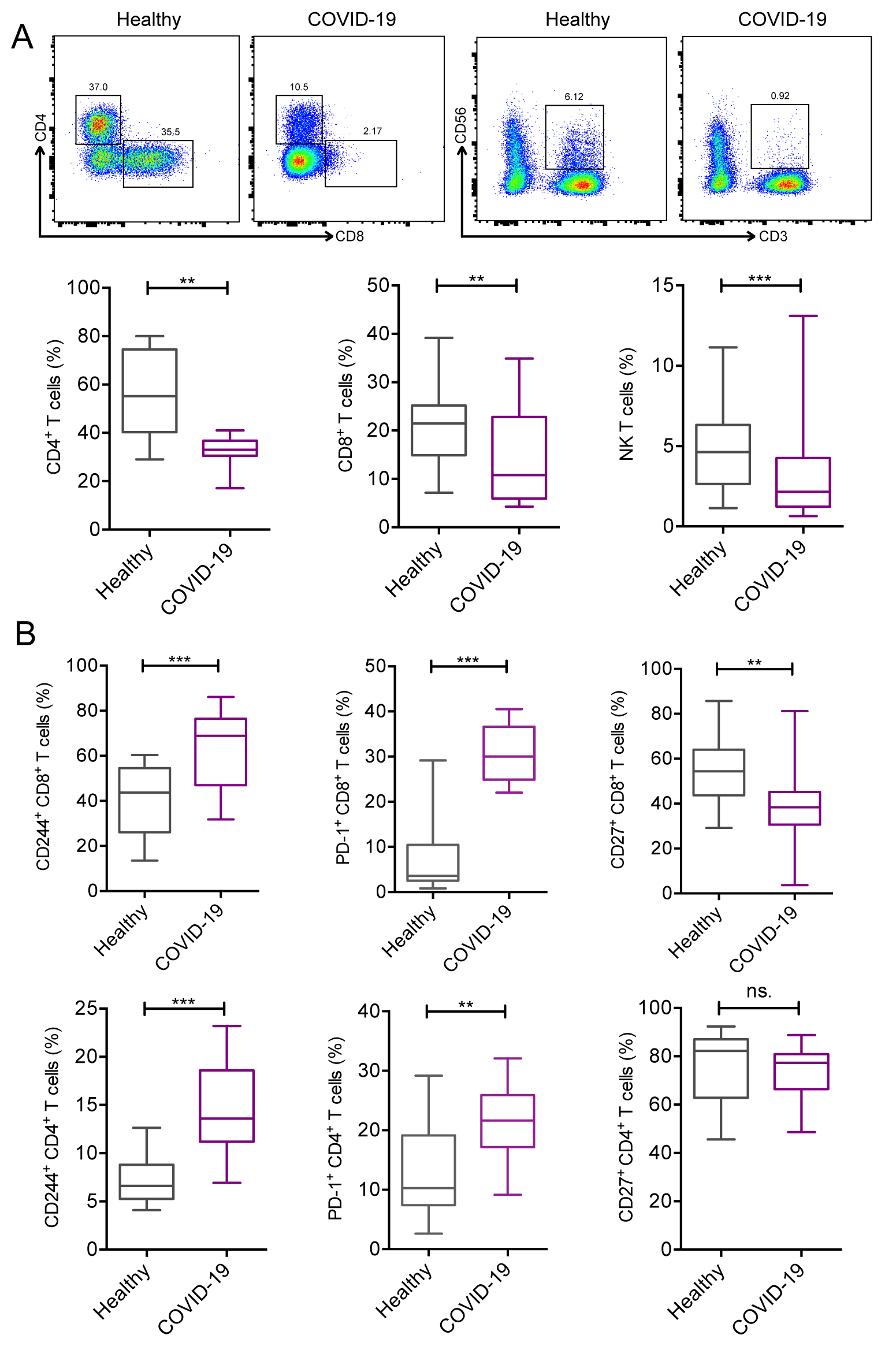

Supplement: Supplementary Figure 3 — COVID-19 patients showed an imbalanced proportion of T cell subsets and increased levels of regulatory molecules in peripheral blood T cells. (A) Percentages of CD4+T cells, CD8+T cells and NKT cells of total PBMCs and the ratio of CD4+/CD8+ T cells from the healthy controls (n = 37) and patients with COVID-19 (n = 32). (B) Comparisons of cell expression modules of regulatory molecules (CD244, PD-1 and CD27) in CD8+T and CD4+T cells in healthy controls (n = 37) and patients with COVID-19 (n = 32). The level of significance is indicated as follows: ns, not significant; *p <0.05, **p< 0.01, and ***p<0.001. [file Image_3.tif]

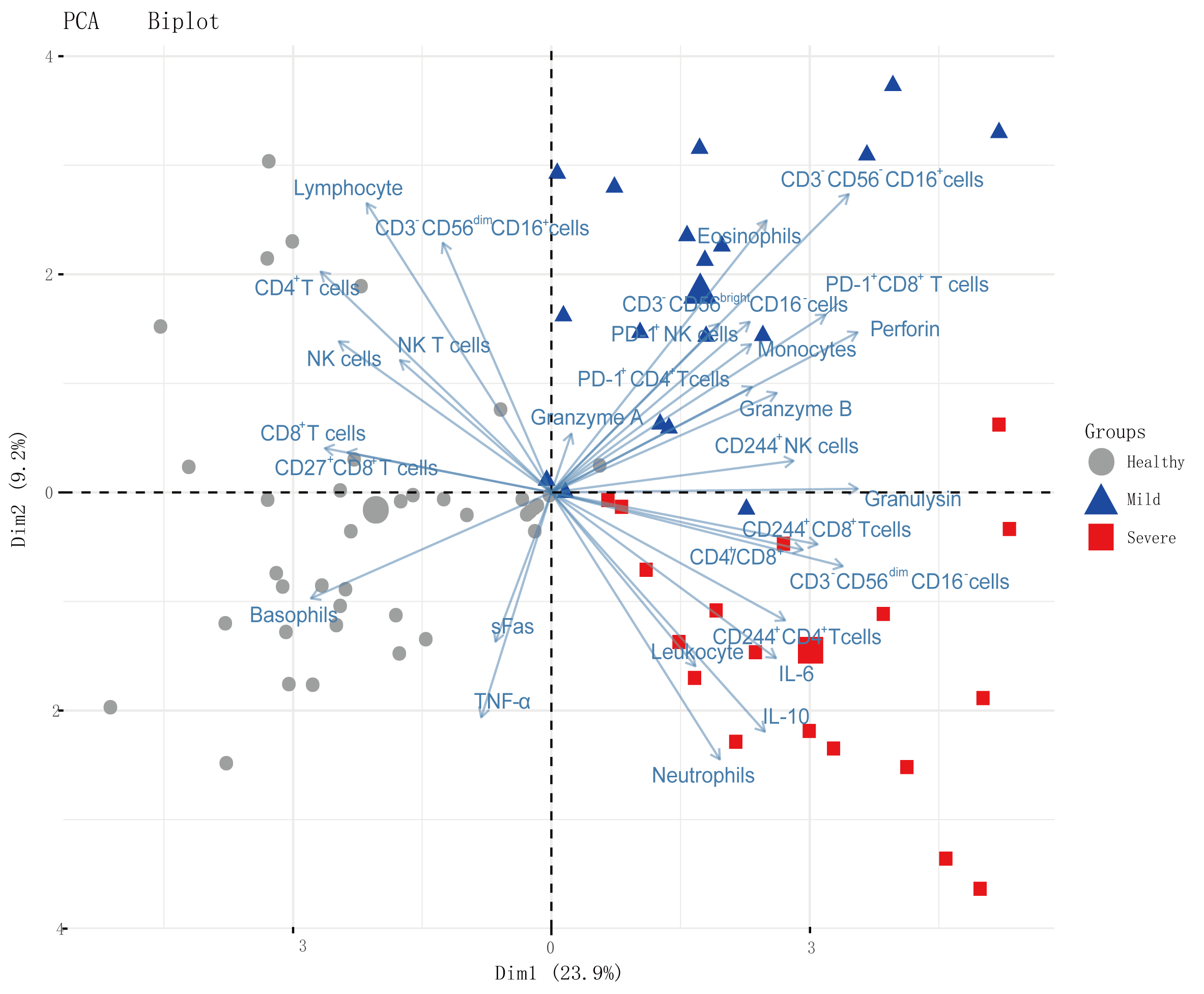

Supplement: Supplementary file 4 [file Image_4.tif]

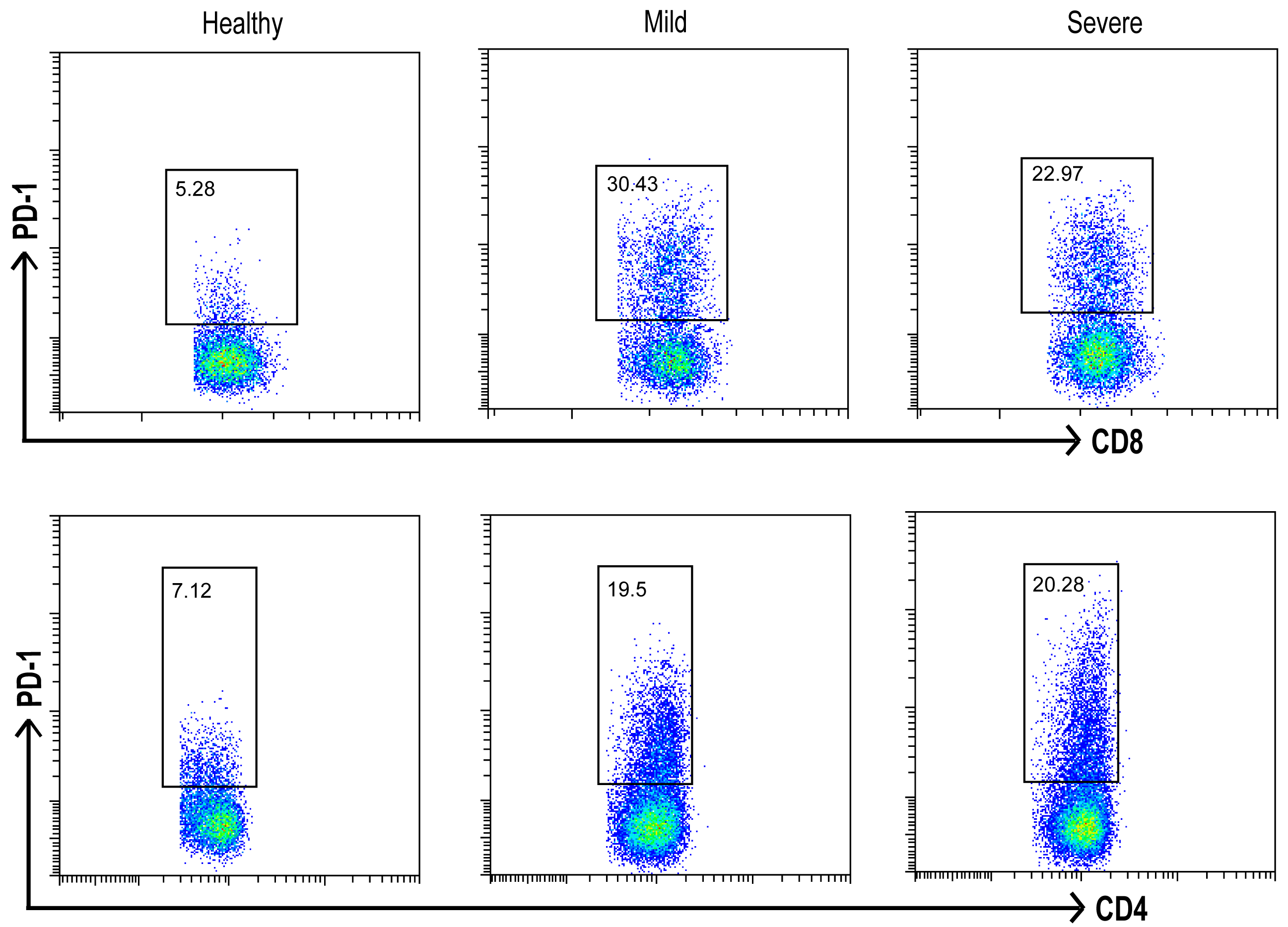

Supplement: Supplementary file 5 [file Image_5.tif]
